# Understanding creativity process through electroencephalography measurement on creativity-related cognitive factors

**DOI:** 10.3389/fnins.2022.951272

**Published:** 2022-11-30

**Authors:** Yuan Yin, Pan Wang, Peter R. N. Childs

**Affiliations:** ^1^Imperial College London, London, United Kingdom; ^2^School of Design, Hong Kong Polytechnic University, Hong Kong, Hong Kong SAR, China

**Keywords:** creativity, cognitive factor, cognitive process, EEG, ERP

## Abstract

**Introduction:**

Neurotechnology approaches, such as electroencephalography (EEG), can aid understanding of the cognitive processes behind creativity.

**Methods:**

To identify and compare the EEG characteristics of creativity-related cognitive factors (remote association, common association, combination, recall, and retrieval), 30 participants were recruited to conduct an EEG induction study.

**Results:**

From the event-related potential (ERP) results and spectral analysis, the study supports that creativity is related to the frontal lobe areas of the brain and common association is an unconscious process.

**Discussion:**

The results help explain why some creativity-related cognitive factors are involved either more or less readily than others in the creative design process from workload aspects. This study identifies the part of the brain that is involved in the combination cognitive factor and detects the ERP results on cognitive factors. This study can be used by designers and researchers to further understand the cognitive processes of creativity.

## Introduction

Creativity can be regarded as the imagining or inventing ability or the cognitive process to associated with producing novel and valuable ideas and products (Plucker et al., 2004; Yin et al., 2021; Harvey and Berry, 2022). Creativity is needed in various areas. Creative students may learn knowledge effectively because they attempt to combine new knowledge with their existing knowledge structures (Goulet-Pelletier and Cousineau, 2022). Creative leaders in an organization may affect the business model innovation and thus affect the performance of companies (Yopan et al., 2022). Creativity is also needed for designing products and architecture, and to find solutions. Designers are able to promote innovative ideas and solutions to deal with problems through the use of creativity.

Understanding how creativity occurs can help people generate more ideas. From the definition of creativity, it can be inferred that creativity is not a simple cultural or social construction; instead, creativity is related to human psychological and cognitive processes (Roca et al., 2021), which happens in a creative person’s mind and is partly out of conscious control (Brem and Puente-Díaz, 2020). Thus, researchers have tried to connect the cognitive process with creativity and concerned on understanding the cognitive process in creativity to understand creativity (Miller, 2014).

Creativity can be achieved through creative idea generation and convergent thinking processes. The former is mainly based on divergent thinking which is about finding different creative solutions to problems while the latter is mainly about insights of problem solving (Benedek and Fink, 2019). Most times, creative ideas are generated based on the combination of the two processes (Childs et al., 2022). This has been supported from existing cognitive models of creativity such as four-stage models (Basadur and Gelade, 2005; Miller, 2014), dual-process models (Gabora, 2010; Nijstad et al., 2010; Gabora et al., 2014), tripartite-process models (Leschziner and Brett, 2019), and cognitive-factor process models (Bhattacharya and Petsche, 2005).

From the models, some cognitive factors which are related with creativity have been indicated. To be specific, semantic memory (Beaty et al., 2020), episodic memory (Madore et al., 2015; Benedek and Fink, 2019), association (Benedek et al., 2020), and combination (Wan and Chiu, 2002) have been identified as the cognitive components of creativity process. The relations between the cognitive factors and creativity have thus been identified. Researchers have reported that semantic memory can contribute to creativity as it can provide facts and concepts, which can be combined to generate new ideas, to support creativity. Also, semantic memory is helpful in associating low-related concepts (Beaty et al., 2014). Episodic memory is helpful in creativity as it is related to stimulating previous memory and reconstructing the details of previous events. This retrieving and combining of previous memory processes can stimulate imagination (Madore et al., 2015). More findings were also promoted such as highly creative people are more likely to utilize remote association during a creativity process (Olson et al., 2021).

Researchers have started to use neurotechnology to investigate the neurological processes behind creativity. However, existing research mainly identified which parts of the brain are involved in creativity, which brain waves are implicated, and which cognitive factors are related to creativity (Benedek and Fink, 2019). Few studies have investigated the various EEG-related characteristics of creativity-related cognitive factors, such as event-related potential (ERP). Therefore, to address the gap in the existing research, this study aims to identify the EEG-related characteristics of creativity-related cognitive-factor process and then compare the differences that these creativity-related cognitive factors have when studied through EEG.

## Literature review

Cognitive processes are people’s thinking processes which bring influences to a behavior (Budiana, 2014). In the creative process, information about the creative task is transformed, stored, recovered and otherwise used in the brain, which indicated that creativity process can be considered a cognitive process in the mind (Hollan et al., 2000). As an element of cognitive process, “cognitive factor” is the immaterial thinking phenomenon in the mind that can affect the thinking process (Alshomrani and Akram, 2013). One of the reasons why people use the creative process to different extents of success is because of different designers’ creative process strategies on cognitive factors utilization, which have different impacts on the creative process (Abraham, 2013). Therefore, to better understand the role of cognition during the creative process, it is worthwhile to investigate the performance of these creativity-related cognitive factors.

A few cognitive factors have been identified as having relation with creative processes, such as memory processing where people consciously search the information in their mind (Beaty et al., 2017; Benedek and Fink, 2019), association processing where people base on two or more concepts to generate more concepts (Nijstad et al., 2010), and combination processing where two or more concepts are mentally synthesized into a new concept (Wan and Chiu, 2002; Carson et al., 2005). In addition to traditional research methodologies, research on applying neurotechnology to identify the relations between cognitive factors and creativity has become increasingly popular.

### Memory

Memory is one of the fundamental elements of creativity (Beaty et al., 2017; Benedek and Fink, 2019). Creativity cannot come *ex nihilo*; instead, it is a process where novel ideas are generated by searching (Fink and Benedek, 2014), interacting (Palmer, 2020) and associating (Benedek and Fink, 2019) existing memories. In some creative thinking processes, before generating new ideas, designers may consciously search the information in their mind to extract useful information. As the source of new ideas, memory has been identified with the activity of amygdale by functional magnetic resonance imaging (fMRI) (Dinar et al., 2015). Alpha (Ali et al., 2022), theta (Wang et al., 2022), and gamma waves (Sharpe and Mahmud, 2020) were detected to be related to the memory processes. It is important to note that memory processes can be divided into two types. One process is encoding memories, and the other is extracting useful memories. In this study, the process referred to is the extracting memory process rather than the encoding process. funding Memories can be further divided into long-term memory (LTM), short-term memory (STM), and sense memory. Sense memory is the information that is acquired through hearing, vision, touch, and other senses (Di Benedetto, 2007). Few research studies have focused on identifying the relations between sense memory and creativity because sense memory is an unpredictable process and is hard to be controlled in labs.

Short-term memory has a longer processing time (a few seconds or a few minutes) than sense memory. Unlike sense memory, which works unconsciously, STM allows people to repeatedly and consciously recall facts and events (Norris, 2017). STM is involved in creative processes because creativity processes require to store information temporarily (Mao et al., 2020). Gubbels et al. (2017) identified the relationship between STM, analytical ability, and creativity. They asked children to look at 20 graphics and remember them. Then, participants were asked to write down a description of each graphic based on their memory. In another task, participants were asked to write down 10 words they heard and then rewrite them based on what they could remember. The results showed that (visual and language) STM affects the analytical ability and the level of creativity. The reason why it is hard to test STM ability in a creativity task is that participants were not able to report their STM processes in a creative task as the reporting process will interrupt the STM process. This is the reason why neuroscience technologies are needed for reporting tasks. With the help of neuroscience methods, it is found that STM is positively associated with delta and theta waves and negatively associated with alpha waves (Trammell et al., 2017).

Long-term memory is the memory that has been stored in the brain for a long time (Norris, 2017). LTM has been associated with creativity because LTM includes information about previous knowledge. This knowledge can be used to create ideas that are related to creativity tasks (Goldschmidt, 1995). LTM can be divided into declarative memory and non-declarative memory. Researchers have found that creativity is related with LTM, especially declarative memory (Benedek et al., 2020). Declarative memory is the memory that people can access consciously. It can be further divided into semantic memory and episodic memory. Semantic memory is the memory of facts which will not be changed or limited by time and space. For example, the current capital of China is Beijing. Semantic memory is considered to have a relationship with creativity for it can provide information about facts and concepts, acting as a source of creativity to generate new ideas (Kenett and Faust, 2019). Semantic memory can also support the association of related concepts (Volle, 2018). The association process may also be helpful to generate creative ideas (Huang et al., 2015; Beaty et al., 2020).

Episodic memory is the memory that an individual experiences in a specific time and location, such as information on daily life; for example, the memory “today, I drank a glass milk in the morning.” Episodic memory can support the generation of creative ideas (Madore et al., 2016) because it allows people to stimulate their memories of previous events (Benedek and Fink, 2019) or provide details of previous events as the creativity stimulation (Madore et al., 2015). Moreover, in the episodic memory process, people do not simply search their memory and select useful sources individually. Episodic memory processes also restructure memories in the brain. In other words, the event that people recall is not the original event that people went through; instead, it has been processed by the brain’s episodic memory process. The retrieval and reconstruction processes act as a source of creativity and helps to stimulate the imagination (Beaty et al., 2020).

Researchers have investigated the application of neurotechnology to identify the relationship between semantic or episodic memory and creativity, some of whom have focused on the neural structure of the brain. When engaged in the creative process, the default mode network (DMN), which is related to semantic and episodic memory, will be activated (Benedek and Fink, 2019). With the help of fMRI, semantic memory in a creative process was further identified to be related to the left angular gyrus, left inferior parietal lobule and posterior cingulate cortex, while episodic memory in a creative process was further identified to be related to left parahippocampal gyrus and right inferior parietal lobule (Beaty et al., 2020).

For convenience, the following terms in this study are used: “retrieval” is used to represent the episodic memory process, as episodic memory is related to the retrieval of a previous event; “recall” is used to represent semantic memory process, as semantic memory is related to recalling the knowledge of a fact.

### Association

Many researchers have realized the importance role of the cognitive process of association in creativity (Guilford, 1956; Finke et al., 1992; Wu et al., 2021; Yin et al., 2022). Association comes in the forms of remote and common associations (Benedek et al., 2020). Remote association is the ability to associate unrelated concepts while common association is the ability to associate related concepts. Remote association has a positive effect on creativity (Liu, 2016; Benedek et al., 2020). The activity of alpha waves was identified to be related with remote association (Fink et al., 2009). Researchers have also tried to identify which parts of the brain are related with remote association. However, studies on the involvement of remote association have not found consistent results. Fink et al. (2009) suggested that the left frontal lobe was active when people make use of remote association in creative processes (Purcell and Gero, 1998). Stevens and Zabelina (2019) supported the view that the left temporal lobe is related to remote association processes in creativity, whereas Jung-Beeman (2005) thought it was the right temporal lobe that relates to remote association processes. Common association can also contribute to the creative ideas. The differences between remote association and common association are on the creative ideas quantity (Purcell and Gero, 1998). Fewer creative responses were generated through common association processes compared to remote association processes. In addition, compared with remote association, the activation levels of brain and the alpha waves during common association process is weak (Purcell and Gero, 1998; Stevens and Zabelina, 2020).

### Combination

Combination is also considered to be one of the cognitive processes involved in creativity. Combination ability is related to attention and LTM. When people have broad attention, they have a better chance to combine relative information with new concepts (Carson et al., 2005). Additionally, combining concepts is one of the operations controlled by LTM (Simon and Simon, 1978), especially with regard to episodic memory (Kenett and Faust, 2019), because they are the source from which a combination can lead to a concept. Researchers have applied quantitative studies to identify the relations between combination process and creativity (Wan and Chiu, 2002). Results have shown that the creativity score of novel combination tasks is higher than that of common combination tasks. However, few studies so far have applied neuroscientific methods to identify the relationship between combination and creativity.

### Study aims

Different neurophysiological characteristics of creativity-related cognitive factors (recall, retrieval, combination, and association) have been identified. However, the existing studies mainly identified the relationship between cognitive factors and creativity singly, focusing on which part of the brain is activated in the process and which type of wavebands are related. Other neurophysiological characteristics, such as event-related potentials (ERPs), have not been fully studied. Also, the neurophysiological characteristics of creativity-related combination processing have not been studied in detail. Therefore, this study aims to understand the EEG characteristics of creativity-related cognitive factors (recall, retrieval, combination, and association) in this way to better understanding the creativity process from neurophysiological levels.

## Methodology

The following five EEG-induced tasks were conducted to better understand the different EEG characteristics that occur during the cognitive processes of remote association, common association, retrieval, recall, and combination.

### Participants

In total, 30 right-handed Chinese participants (15 female, 15 male, aged 20–25), experienced in industrial or product design, were recruited. Their creativity levels were reported from the Epstein Creativity Competencies Inventory for Individuals (ECCI-i), which is a 28-items 5-point Likert-type scale labeled from Strongly Disagree (1 point), Disagree (2 point), Neither (2 point), Agree (4 point) to Strongly Agree (5 point; Epstein et al., 2008). All participants’ ECCI-i scores were over 120, which indicated that the participants have a strong potential to have creative ability (Epstein, 2000).

Before the study, all participants were ensured through self-reporting to have normal or corrected-to-normal vision and have no diagnosed psychiatric disorders, color blindness or other barriers to using computers. Also, it was ensured that the participants did not ingest any caffeine, unprescribed medication or alcohol in the previous day before taking part in this study. After the study, all participants self-reported that they had not seen the design tasks finished in this study before and had expressed their ideas clearly. This study was approved by the local ethics committee of the first author (reference number: 20IC6227).

### Task and procedure

**FIGURE 1:**
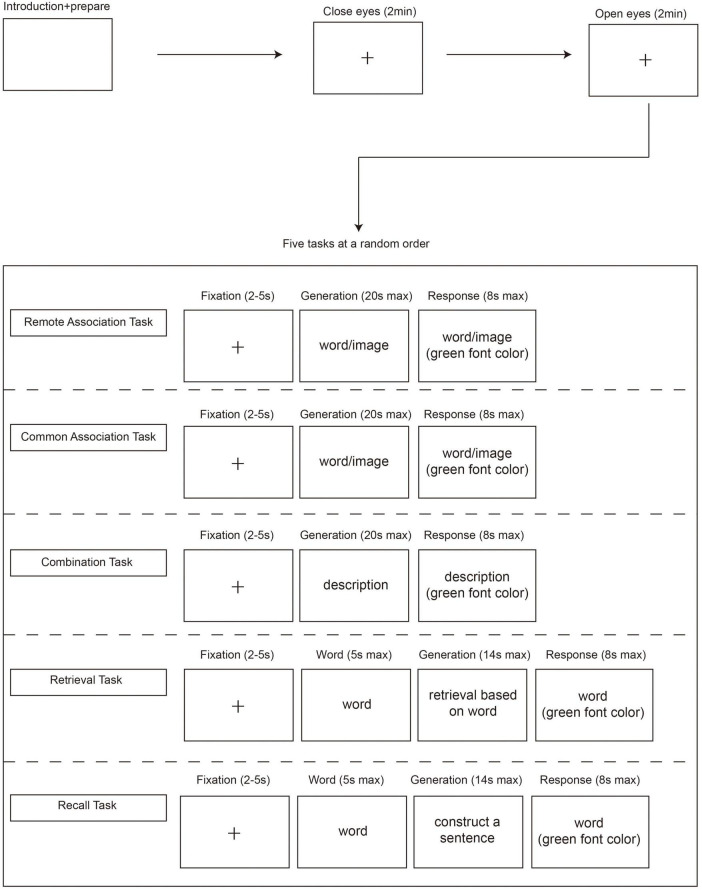
Study procedure. After introducing the tasks, the electroencephalography (EEG) devices were worn. Participants first opened or closed their eyes for 2 min. Then, the five cognitive-factor induction tasks (each one tailored to remote association, common association, retrieval, combination, and recall) were conducted in random order. The entire study lasted around 1 h. Participants can have a 5-min rest after each induction task.

The study included five induction tasks to test participants’ remote association, common association, combination, retrieval, and recall ability. The procedure of the study is shown in Figure 1.

#### Remote association

##### Task

The AUT was used to measure the participants’ remote association ability (Wilson et al., 1953; Purcell and Gero, 1998; Fink et al., 2007; Schwab et al., 2014). To be specific, participants were asked to think of a remotely related use for each of a selection of everyday objects (for example, umbrella – boat for animals). Since participants may be unfamiliar with the concept of “a remotely related use,” the study used the expression “a use of the given object that only few people would think of” instead (Purcell and Gero, 1998).

In the AUT task, 15 everyday object words and 15 everyday object graphics were presented to participants (all words and graphics are listed in Supplementary Appendix 1). Each word or graphic was presented once in a random order. The descriptions of the words and graphics were collected from Stevens and Zabelina (2020). The corresponding graphics were collected using the BaiduImage search engine, a common image search engine in China. Images that represented the object words were selected. The sizes of the images were resized to 500 × 500 pixels.

The reason why both words and graphics were selected in this task is that ideas may emerge in the brain in a form of an image or text. Including both words and graphics can, thus, reduce the bias generated from different emerging forms. Because this study aimed to identify the creativity-related remote association ability, in the analysis, the words and graphics results were analyzed together. In this case, the different EEG characteristics generated from different thinking forms (images or words) were removed from the EEG characteristics of remote association.

##### Procedure

Each of the 30 trials began with a black fixation cross on a light gray background. The fixation cross appeared on the screen for 2–5 s. Then, a word or graphic was displayed in the middle of the screen. Participants had up to 20 s to think of a use of the given object that only few people would think of but not verbalize. If a solution was found before the timeout, participants could use the space key on the keyboard and jump to the response interface. If the 20 s ran out, the interface would jump to the response interface automatically. In the response interface, a green text was displayed to remind participants to vocalize their answers within 8 s. The protocol for this task is displayed in Figure 1. This task took about 15 min to complete.

#### Common association

##### Task

In the common association task, participants were asked to think of a highly related characteristic for each object (for example, shoes –paired). Since the participants may have been unfamiliar with “a highly related characteristic,” the “first characteristic that came to mind and that most people would think of” was used to represent a highly related characteristic (Purcell and Gero, 1998).

There were 30 trials in total. Among the 30 trials, participants saw 15 words and 15 graphics (all words and graphics are listed in Supplementary Appendix 2; Purcell and Gero, 1998; Stevens and Zabelina, 2020). The words and graphics collection method in the common association task was the same as that of the AUT (section “Remote association”). Each word or graphic was presented once in the task in a random order.

##### Procedure

Each of the 30 trials began with a black fixation cross on a light gray background. The fixation cross appeared on the screen for 2–5 s. Then, a word or graphic was displayed in the middle of the screen. Participants had up to 20 s to report the first characteristic comes to mind that most people will think of but not verbalize. If a solution was found before the timeout, participants could use the space key on the computer keyboard and jump to the response interface. If the 20 s ran out, the interface would jump to the response interface automatically. In the response interface, a green text was displayed to remind participants to vocalize their answers within 8 s. The protocol for this task is displayed in Figure 1. This task took about 15 min to complete.

#### Combination

##### Task

Since in creative process, both novel combination and ordinary conceptual combination may happen, the studies tested both of the conditions. The average EEG results from novel combination and ordinary combination was the EEG results of combination sub-process during creativity. The participants’ combination ability in creative process was tested by adjusting the protocol from Wan and Chiu (2002). In this task, participants were asked to complete nine ordinary conceptual combination trials and nine novel combination trials. The trials were obtained from Hampton (1997) and Wan and Chiu (2002). All tasks are listed in Supplementary Appendix 3. Each trial was presented once in the task. The task included 18 trials. The order of the presentation was random.

In each novel conceptual combination trial, participants were asked to combine a pair of objects whose attributes were incompatible. The results of each combination trial should be an object that satisfied the trial description. Since the two concepts were incompatible, the intersection of the two concepts has not existed in real life. In other words, the result generated by participants was something not existing in real life. Therefore, the result could be considered a novel combination of two disparate concepts.

In each ordinary conceptual combination trial, participants were asked to combine a pair of objects whose attributes were compatible. The results of each combination trial should be an object that satisfies the trial description. Since the two concepts were compatible, the intersection of the two concepts exists in real life. The result generated by participants was something that exists in real life. Therefore, the result could be considered an ordinary combination of two related concepts.

##### Procedure

Each of the 18 trials began with a black fixation cross on a light gray background. Then, the 18 trials were displayed and remained on the screen for up to 20 s. During this period, participants were asked to “think of an object that satisfies the trial description but not verbalize it.” If a solution was found before the timeout, participants could use the space key on the computer keyboard and jump to the response interface. If the 20 s ran out, the interface would jump to the response interface automatically. In the response interface, the text would change to green, which reminded the participants to vocalize their response in 8 s. The protocol for this task is displayed in Figure 1. This task took about 10 min to complete.

#### Retrieval

##### Task

In this creativity-related retrieval-ability task, participants were asked to creatively retrieve stored information based on the given words. Since participants may not know the meaning of the “creatively retrieve,” the study used “retrieve brain-stored information that few people may retrieve based on the given words” to instead. The tasks were obtained from Beaty et al. (2020) and are listed in Supplementary Appendix 4. There were 30 trials and each task was presented once.

##### Protocol

Each of the 30 trials began with a black fixation cross on a light gray background. Then a word was displayed in the middle of the screen and participants were asked to identify the word in 5 s but not verbalize it. If the word was recognized before timeout, participants could use the space key on the keyboard and jump to the generation interface. If the 5 s ran out, the interface would jump to the generation interface automatically.

In the generation interface, participants were asked to retrieve brain-stored information that few people may retrieve based on the given words in 14 s but not verbalize it. If a solution was found before timeout, participants could use the space key on the keyboard and jump to the response interface. If the 14 s ran out, the interface would jump to the response interface automatically. In the response interface, the text would change to green, which reminded participants to vocalize their response in 8 s. The protocol for this task is displayed in Figure 1. This task took about 10 min to complete.

#### Recall

##### Task

In this creativity-related recall-ability task, participants were asked to construct a creative sentence based on a given word. Since participants may not know the meaning of the creative sentence, the study used “construct a sentence that few people can think of based on a given word” to instead. The tasks were obtained from Beaty et al. (2020) and are listed in Supplementary Appendix 5. There were 30 trials and each task was presented once.

##### Protocol

The protocol of the recall task was the same as that of retrieval task, apart from, in the generation interface, participants were asked to construct a sentence that few people can think of based on a given word in 14 s but not verbalize it.

#### General procedure

An information sheet and a consent form were first sent to participants before the EEG study. Participants could ask any questions for clarification. If there were no questions, they could sign the consent form. Then, participants were instructed on how to perform the remote association, common association, retrieval, combination and recall tasks. After what was expected in each task was explained to the participants, the EEG device was put on with the help of the researchers. Before the EEG study started, participants were told that they could rest when a task was finished or that they could take off the EEG device to rest. Participants were told that they could move their eyes freely while speaking, but they needed to keep themselves still once the next fixation cross appeared (Stevens and Zabelina, 2020).

At the beginning of the EEG study, participants were asked to maintain a resting state. Participants closed their eyes for a duration of 2 min, after which they opened their eyes for a duration of 2 min. Then, the five cognitive-factor induction tasks (each testing remote association, common association, retrieval, combination or recall ability) were conducted in a random order. The entire study lasted around 1 h. The procedure is shown in Figure 1.

### Electroencephalography recording and equipment

**FIGURE 2:**
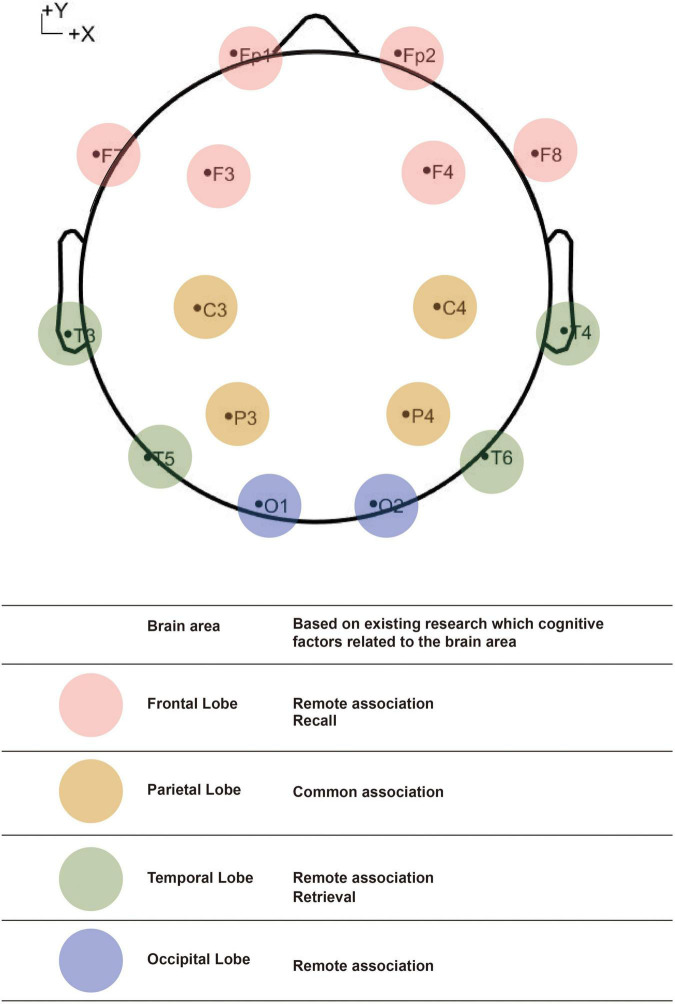
The position of the 16 scalps and the relations between the 16 channels and brain areas, and which cognitive factors related to the brain areas have been mentioned.

A medical-grade EEG device, the Neurofax EEG-9200 system, was used to record the EEG signals (NIHON KOHDEN, Tokyo, Japan). The Neurofax EEG-9200 system includes 16 scalp and 2 mastoid Ag/AgCl electrodes mounted according to the 10/20 system (Figure 2). Also, it includes an EEG measurement system, an amplifier and an EEG result viewing software. The impedances of all the EEG channels were below 5 kΩ. The data were sampled at 1,000 Hz. The EEG tasks were generated and presented with the help of E-Prime 3.0. All tasks were presented on a computer screen (35.89 × 24.71 cm with a resolution of 2,560 × 1,600). The data were collected and stored in the Neurofax EEG-9200 system.

### Why 16 channels

Because the EEG electrodes are relatively far from the neurons where the signals are generated, EEG has a relatively low spatial resolution (Srinivasan, 1999). Therefore, increasing the number of electrodes may only provide diminishing returns in terms of EEG data acquisition. In other words, with more electrodes, the correlated signals may tend to interfere with adjacent channels. Statistical methods are often used to combine signals of interest coming from multiple channels into a single signal. In other words, increasing the spatial sampling density (channel quantity) means more channels will be included in that cluster, instead of increasing the efficiency of the data. Therefore, more channels may not generate effective EEG data. A further issue with this is that more channels increase the processing time for the data that are stored and analyzed.

According to previous research, the areas of the brain that relate to creativity-related cognitive processes (remote association, common association, retrieval, combination, and recall) have been found. The results are visualized in Figure 2. Remote association was related to the right temporal lobe (Jung-Beeman, 2005), left frontal lobe (Purcell and Gero, 1998; Fink et al., 2009), and occipital cortex (Boccia et al., 2015). Common association was related to the inferior parietal lobe (Benedek et al., 2014). Retrieval was related to the medial temporal lobe (Madore et al., 2016; Beaty et al., 2020). Recall was related to the frontopolar cortex (Green, 2016; Beaty et al., 2020). There was no research to identify which areas of the brain were stimulated by combinations of these cognitive processes.

Compared with the finding areas and the areas covered by the 16 EEG channels, most of the identified areas were covered. Thus, more channels may not be necessary. To be specific, channels Fp1/Fp2/F7/F8/F3/F4 report signals on the frontal lobe, C3/C4/P3/P4 report signals on the parietal lobe, T3/T4/T5/T6 report signals on the temporal lobe and O1/O2 report signals on the occipital lobe. Considering that the study is also interested in potential hemispheric differences, midline electrodes such as FZ, CZ, and PZ, were not included (Schwab et al., 2014).

### Data pre-process

The MATLAB R2018b (The MathWorks Inc., Natick, MA, United States) plugin EEGLAB was used to analyze the signals. A 50 Hz notch filter was applied to negate the interference of the electrical mains. Then, the signals were passed through a band-pass filter with a pass-band of 0.1–100 Hz (Zarjam et al., 2011; Schwab et al., 2014). The reference electrodes were placed on the left and right mastoid processes.

The study then compared the ERP and active brain areas of the five EEG events (remote association, common association, combination, retrieval, and recall) by marking and extracting them from the EEG signals. The ERP subject averages for each event were the averaged results of all participants and all event-related task trials.

## Results

### Spectral analysis

**FIGURE 3:**
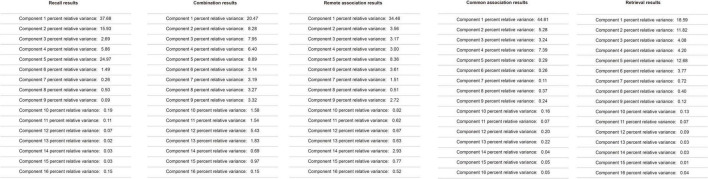
Electroencephalography spectral analysis results for remote association, common association, recall, retrieval, and combination induction tasks.

Spectral analysis was conducted for each event. The percent relative variance, sometimes called the relative variance, was calculated. The spectral results of each event are shown in Figure 3. Some previous studies have suggested that percent relative variance can be used to define the effect of a particular variable on the whole condition (Hermance, 2013). In EEG, the component percent relative variance was used to represent the contribution of a specific component on a particular channel or the whole channels (Delorme and Makeig, 2004). Therefore, in this study, the percent relative variance defines the effect that a particular component of EEG on the whole EEG channel or a particular individual channel.

**FIGURE 4:**
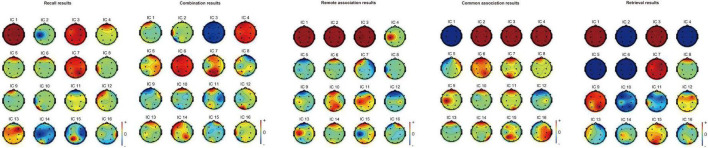
ICA results for remote association, common association, recall, retrieval, and combination induction task.

The component X (1 - 16) from the spectral analysis is the same component from the independent component analysis (ICA) results. The ICA results of each event are shown in Figure 4. Since the percent relative variance reported the effect that a particular component of EEG on the EEG channels, to identify which brain areas were related to a specific cognitive-factor event, in each specific cognitive-factor event, the component percent variances were ranked. Then, the highest component percent variance that related to specific brain areas was used as the cue to identify which brain areas were activated in the specific cognitive-factor event.

The results demonstrate that the cognitive processes of remote association, common association, recall and retrieval mainly relate to the frontal lobe (Fp1 and Fp2 channels; based on component 5, component 8, component 5, and component 8, respectively). The combination process mainly related to the left frontal lobe (Fp1 channel; based on component 1).

### ERPS results

The ERPS results of each event were analyzed based on the related activated brain area EEG channels from the spectral results. Specifically, remote association, common association, retrieval and recall events are based on the Fp1 and Fp2 channels. The combination events are based on the Fp1 channel.

**FIGURE 5:**
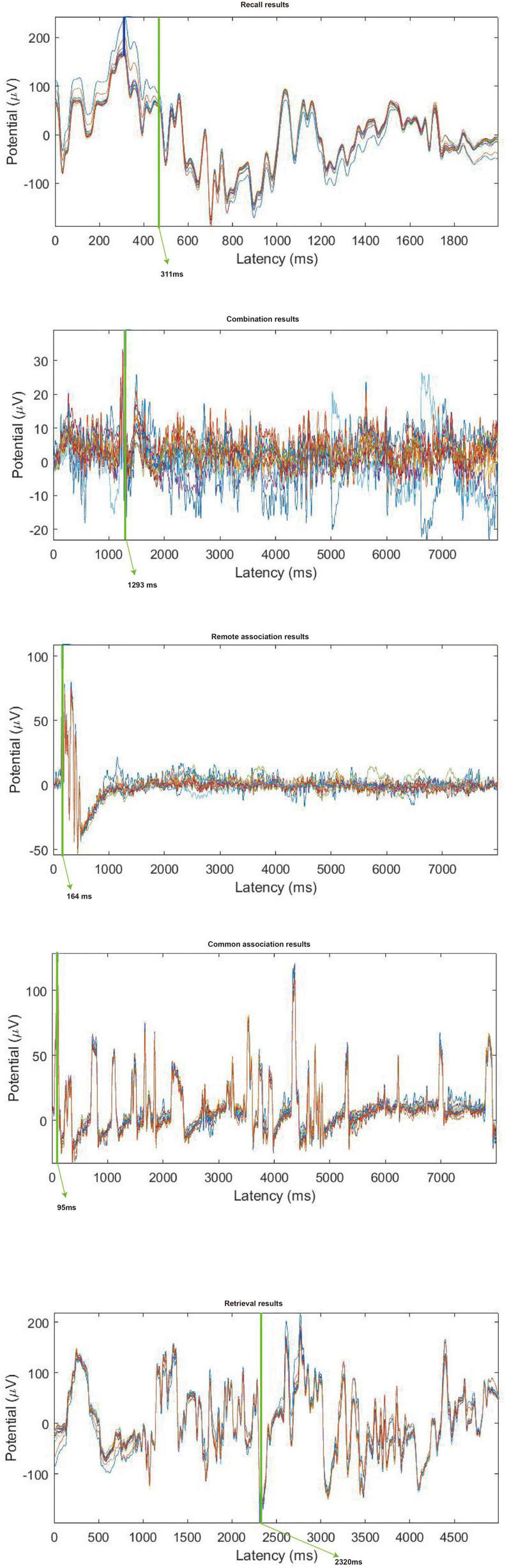
ERPS results for remote association, common association, recall, retrieval, and combination induction tasks. ERPS results for remote association, common association, recall, retrieval, and combination induction tasks.

The results have been presented in Figure 5. The highest ERP for the remote association event was generated at 164 ms. The highest ERP for the ERP was generated at 95 ms. The highest ERP for the combination event was generated at 1,293 ms. The highest ERP for the retrieval event was generated at 2,320 ms. The highest ERP for the recall event was generated at 311 ms.

## Discussion

### Comparing active brain areas with existing research

Although the location information of EEG is not that accurate, it does, to some extent, reflect the active brain areas and thus is compared to existing research.

#### Remote association

The study indicated that in creative cognitive activities, remote association is related to the frontal lobe brain area, which is consistent with the findings of some existing studies (Purcell and Gero, 1998; Fink et al., 2009). However, the results were inconsistent with some other studies, which indicated that remote association was related to the left temporal lobe (Stevens and Zabelina, 2019), or the right temporal lobe (Jung-Beeman, 2005). The difference may be because LTM (semantic and episodic memory) is related to association (Purcell and Gero, 1998; De Dreu et al., 2012; Menashe et al., 2020). Semantic memory is related to the activity of the frontopolar cortex (Beaty et al., 2020), while episodic memory is related to the activity of the temporal lobe (Madore et al., 2015). When identifying the areas of the brain that are activated by remote association, it was found that similar areas may also be activated by the LTM. The identified brain areas active during remote association may also include the brain areas active when a person accesses their LTM. Therefore, there are various areas of the brain that are active during remote association.

In addition, as mentioned before, EEG has a low spatial resolution and a high temporal resolution. Some other neurotechnology methods, such as fMRI has a high spatial resolution and a low temporal resolution. This means using different neurotechnology, the location results may be different. For example, the active brain location of remote association was left frontal lobe when collecting data from using fMRI (Fink et al., 2009), while it was left temporal lobe when using EEG to collect data (Stevens and Zabelina, 2019).

In addition, the results may also relate to induction tasks. On the one hand, this study used AUT task as the induction task while some other researchers may use remote association test (RAT) where participants were asked to find a solution based on the given words without time limited and said aloud the most original word-association (Razumnikova, 2007). On the other hand, the existing studies did not distinguish the different induction forms (graphic and text). The results of this study were from both graphic and text induction while the results of other studies were more likely from the text induction.

#### Common association

The location of the brain stimulated by common association is consistent with findings from the existing studies, which locate this stimulation in the frontal lobe (Jung-Beeman, 2005).

#### Combination

To date, no study has applied EEG to identify the parts of the brain stimulated by combinations cognitive factors in creative activities. Therefore, this study first proposes that these combinations of cognitive factors in creative activities are related to the left frontal lobe.

#### Retrieval

The study found that cognitive processing of retrieval is located in the frontal lobe. This is different from existing studies, which found that retrieval was related to the medial temporal lobe (Beaty et al., 2020). This difference might be because of the reliance on fMRI that was noted in the Beaty et al. (2020) study. In other words, applying different neurotechnological tools may be the reason for why the different results occurred.

Additionally, apart from the similar reasons mentioned in section “Remote association,” there is another possible explanation. The highest component percent variance that related to a specific brain area was used to identify which brain areas were activated during the specific cognitive factor event. The area related to retrieval tasks was identified based on component 8. However, other components (such as component 16) also indicated that retrieval may be related to the temporal lobe area. The relative variance of component 16 was to a lesser degree to that of component 8, therefore it was less likely to represent the brain area stimulated by retrieval; but, this lesser possibility does not equate that retrieval has no relation to the temporal lobe.

#### Recall

The frontal lobe was found to be stimulated by the recall activities. This is similar to existing studies which pointed out that recall was related to the frontal lobe area.

#### Comparison among the five events

From the results, it can be seen that all five cognitive factor events were related to the frontal lobe. The recall and combination events results were even more detailed, indicating that they are related to the left frontal lobe area. This is acceptable because researchers have mentioned that creativity is related to the left brain (Zaidel, 2014). However, some researchers pointed out that the right brain still exerts some control over creativity (Sawyer, 2011). Our study may explain why this controversy exists. As mentioned before, existing research mainly focused on a specific creativity-related cognitive factor. The activated brain areas of different creativity-related cognitive factors may be different. People who use recall and combination events to drive their creative processes may be more likely to provide evidence that creativity is related to the left brain, while people who use other cognitive factors to drive creativity may be seen to have other areas of their brain stimulated. In addition, although the study summarized and compared the location results, considering the fact that the EEG has a low spatial resolution, the results on the location may be less reliable.

### Discussion on event-related potential results

Event-related potential can quantitatively reflect the brain’s response to a specific cognitive event (Sa, 2005). From the ERP results, a few findings can be summarized.

#### Comparison among five events

##### Workload

Event-related potentials can report the evoked time of the events and the cognitive load of the events. The more evoked time used, the greater cognitive load may be needed, which indicates that this event is hard to be achieved (Imbir et al., 2021). The results of this study indicated that retrieval requires more evoked time, followed by combination, then remote association, then recall and, finally, common association. This means retrieval needs a higher cognitive workload and is harder than combination, remote association, recall or common association to be evoked.

This, to some extent, explains why existing research is more likely to focus on detecting the relation between association and creativity or recall and creativity. The two cognitive factors are more easily able to be evoked in the process of creativity and are therefore more likely to be made use of by designers and researchers.

##### Retrieval is a more complex process

After the highest ERPs among the five cognitive-factor events were compared, it was found that ERPs of retrieval was the largest. This means that, among the five cognitive-factor events, retrieval needs the longest time to be evoked. In other words, there is more possibility that other cognitive factors may interrupt the retrieval process and the mind will switch from retrieval to the interrupting cognitive factor.

Highly creative people can force themselves to maintain retrieval processing consciously, whereas low creativity people may not be able to control this process; thus, interruption happens. Since retrieval can bring more creative sources to people, the quality of a person’s creative abilities may be reduced without retrieval processes (Cetinic and She, 2022). This further indicates that retrieval is a conscious process that can be controlled and trained by humans. This suggests that when people realize that their cognitive processes do not include retrieval and that highly creative people are more likely to have the retrieval process, they may attempt to develop their retrieval processing to improve the quality of their creativity.

#### Comparison between remote association and common association

The highest ERP result for remote association was 162 ms, while that of common association was 95 ms. Therefore, the results demonstrate that the participants had a faster response in the common association task than in the remote association task. This may be why common association is more likely to occur than remote association (Luft et al., 2018). When a designer plans to use remote association to imagine some creative ideas, common association may occur instead and interrupt the remote association.

#### Comparison between recall and common association

Compared to the ERPs of the recall, it was found that the evoked time for common association was less than that of recall, while the evoked time for remote association was more than that of recall. Although association is a cognitive factor, which is the unit of cognitive processes, the remote association and common association tasks can also be considered as creative cognitive processes on some level. Therefore, this earlier evoked time of common association indicates that common association does not involve the recall process and may be an unconscious process. This further indicates that the cognitive factors with the highest ERPs (remote association, combination and retrieval) are slower than that of recall, which suggests that they may be conscious processes. This hypothesis can be supported by the results of the retrieval test, where the discussion pointed out that retrieval may also be a conscious process.

### Limitation and future research

This study has some limitations. Firstly, this study only recruited 30 Chinese participants. The participants’ culture and ages may have affected the EEG results. Therefore, in the future, more participants from different age groups and cultures should be incorporated.

Secondly, there was an attempt to conduct the study without any external interference (such as motion and noise). However, the study cannot rule out the possibility of spill-over effects completely. In other words, it may be possible that the previous task/trial may have affected later ones. What could be done in future studies is to limit the spill-over effects by presenting the inducted tasks in a random order and presenting the trials in each task in a random order.

Thirdly, the study followed the guided assumption from Beaty et al. (2020) that participants could follow the instructions completely. Also, the study assumed that the EEG recorded in each cognitive factor task represented participants’ cognitive factor ability completely accurately. In other words, the study hypothesized that participants did not thinking of anything that was not related to the cognitive factor tasks and that their thinking of process relied on their cognitive factor-related ability. However, it is hard for researchers to objectively check whether participants did not have thoughts unrelated to the cognitive factor tasks or whether their thought processes solely relied on their cognitive factor-related abilities. Therefore, whether the identified EEG signals solely represented the actual cognitive factor-related EEG signals cannot be ensured. This makes the results less reliable. Even if the study assessed the cognitive factor task results, it would only reflect the participants’ creativity levels; the researchers would still be unsure whether the results were generated from the cognitive factor task-related abilities. Therefore, future studies should add a checking mechanism to increase the accuracy of the EEG quality. For example, after each trail or task, an interface can be displayed and asked participants whether they thought anything that was not related to the cognitive factor tasks and whether they thought relied on their cognitive factor-related ability completely.

Moreover, this research included the ideas (or concepts), which generated from the neural activity in induction processing, would be identical to those from the creative design process, especially for the recall and retrieval induction tasks. Whether the actual condition is the same as what the research hypothesized needs to be further detected. To reduce the bias generated from this limitation, this study adapted the induction tasks that have been done by existing research. Also, all of the selected induction tasks were mentioned to have the ability to identify the relations between a specific cognitive factor and creativity. However, whether the selected induction tasks have this ability was not completed studies and the limitations may still exist.

Finally, the EEG results were collected from a medical-grade, 16 channels EEG device. This EEG device is used for in the industry for medical diagnoses. Therefore, the signal quality is different to EEG devices used in other creativity studies by universities, corporate laboratories and national research institutes, which using the non-clinical-level EEG devices. This higher quality signal can mitigate the limitation of having fewer channels, to some degree. In addition, although from the previous explanation of the diminishing returns of multiple channels, these 16 channels were considered to be enough for the study. However, the researchers could not know if the most effective number of channels is 16 or not. In other words, although the collected results have a tendency toward saturation with a number increasing of channels, whether the saturation point is at 16 channels is not clear. Therefore, studies in the future should experiment with various numbers of channels.

## Conclusion

This study aimed to identify and compare the EEG characteristics for different cognitive factors. To address the study’s aims, 30 participants were recruited to conduct a creative EEG-induced study to collect EEG activity data when the participants were engaged in five factors of cognitive processes (remote association, combination, common association, recall, and retrieval).

This study applied ERPs to identify and compare the EEG characteristics for different cognitive factors. This study can be used to explain why some cognitive factors occur either more or less readily than others when a person is engaged in the process of creativity. This study also investigated and analyzed how combinations of the cognitive factors affected their related brain areas, which has otherwise been an unexplored topic in the literature.

From the analysis of the data, the principal findings are that common association has a lower workload, followed by recall, then remote association, then combination and then retrieval. This may explain why retrieval is less likely to occur in the process of creativity, whereas recall and association are more likely to occur. Also, the study indicated that common association is an unconscious process.

## Data availability statement

The raw data supporting the conclusions of this article will be made available by the authors, upon reasonable request.

## Ethics statement

The studies involving human participants were reviewed and approved by the Science Engineering Technology Research Ethics Committee, Imperial College London. SETREC reference: 20IC6227. The patients/participants provided their written informed consent to participate in this study.

## Author contributions

YY and PC contributed to the conception of the study. YY, PW, and PC contributed to the conception and design of the study. YY conducted the study, performed the analysis, and wrote the manuscript. All authors contributed to manuscript revision, read, and approved the submitted version.
