# Supplementary material for: Understanding creativity process through electroencephalography measurement on creativity-related cognitive factors
Source: Front Neurosci. 2022 Nov 30;16:951272. doi: 10.3389/fnins.2022.951272 (PMC9748076; doi:10.3389/fnins.2022.951272)
Supplement: Supplementary file 1 [file Data_Sheet_1.pdf]

Supplementary Material

1 Appendix A: Material for remote association task

1) Bowl
2) Safety vest
3) Key chain
4) Book
5) Cork
6) Bell
7) Bobby pin
8) Ice pack
9) Paint can
10) Spring
11) Vacuum
12) Cane
13) Chopstick
14) Wheel
15) Spoon
16) Coaster
17) Marker
18) Yarn
19) Bullet
20) Drumstick
21) Water bottle
22) Paper clip
23) Dress
24) Seat belt
25) Lipstick
26) Magnet
27) Thermometer
28) Bowling pin
29) Shaving cream
30) Popsicle stick

2 Appendix B: Material for common association task

1) Brick
2) Plate
3) Rope
4) Toothbrush
5) Broom
6) Fork
7) Flowerpot
8) Tire
9) Shovel
10) Coffee Cup
11) Chair
12) Book
13) Doormat
14) Ruler
15) Belt
16) Garbage Can
17) Rake
18) Curtain
19) Spoon
20) Comb
21) Garden Hose
22) Pillow
23) Umbrella
24) Boots
25) Magazine
26) Dustpan
27) Purse
28) Hammer
29) Pencil
30) Scissors

3 Appendix C: Material for combination task

3.1 Ordinary Conceptual

1) A piece of coat that is also a kind of animal skin
2) A vehicle that is also a kind of fish
3) A food that is also a kind of rock
4) A fruit that is also a kind of human dwelling
5) A bird that is also a kind of kitchen utensil
6) A food flavoring that is also a kind of tool
7) A food flavoring that is also a kind of mineral
8) A computer that is also a kind of teacup
9) A cooking stove that is also a kind of bicycle

3.2 Novel Conceptual

1) A piece of furniture that is also a kind of fruit
2) A weapon that is also a kind of utensil
3) A timer that is also a kind of ornament
4) A food that is also a kind of animal
5) A plant that is also a kind of fuel
6) A vehicle that is also a kind of machine
7) A bird that is also a kind of pet
8) An electronic product that is also a kind of book
9) A lampshade that is also a kind of game

4 Appendix D: Material for retrieval task

1) Tape
2) Seashell
3) Picture frame
4) Saw
5) Shoelaces
6) Flagpole
7) Bobby pin
8) Paint brush
9) Stapler
10) Necktie
11) Vacuum
12) Cane
13) Chopstick
14) Golf ball
15) Spoon
16) Coaster
17) Notebook
18) Tile
19) Wind chime
20) Pen
21) Glue
22) Lollipop stick
23) Pizza cutter
24) Coin
25) Mouse pad
26) Eyeglasses
27) Feather
28) Toilet paper
29) Cotton
30) Wine glass

5 Appendix E: Material for recall task

1) Thumb tack
2) Floss
3) Key chain
4) Book
5) Diaper
6) Bell
7) Carpet
8) Dart
9) Magazine
10) Paper clip
11) Dress
12) Seat belt
13) Eraser
14) Magnet
15) Thermometer
16) Chair
17) Shaving cream
18) Apron
19) Milk carton
20) Light bulb
21) Rubber band
22) Bed sheet
23) Button
24) Padlock
25) Sponge
26) Matches
27) Toothbrush
28) Watch
29) License plate
30) White board
